# Determination of serum alkaline phosphatase reference in healthy children aged 1-18 years

**DOI:** 10.22088/cjim.13.4.749

**Published:** 2022

**Authors:** Mehdi Gholami Bahnemiri, Shivasadat Mirabedini, Parisa Mohammadi, Haniyeh Barmaki, Zohreh Qaffaripour, Masomeh Rezapour, Morteza Alijanpour

**Affiliations:** 1 Department of Clinical Biochemistry, School of Medicine, Babol University of Medical Sciences, Babol, Iran; 2 Department of Clinical Biochemistry and Medical Genetics, Molecular and Cell Biology Research Center, Faculty of Medicine, Mazandaran University of MedicalSciences, Sari, Iran; 3 Department of Clinical Biochemistry, School of Medicine, Urmia University of Medical Sciences, Iran; 4 Traditional Medicine and History of Medical Sciences Research Center, Health Research Institute, Babol University of Medical Sciences, Babol, Iran; 5 Non-Communicable Pediatric Disease Research Center, Health Research Institute, Babol University of Medical Sciences, Babol, Iran; # These authors contributed equally to this work

**Keywords:** Children, Alkaline phosphatase, Reference interval, Biochemistry, Liver function

## Abstract

**Background:**

The growth and development of children affect biochemical variables. This population-based study was designed to evaluate the reference interval for alkaline phosphatase (ALP) routinely measured in the clinical laboratory.

**Methods:**

For this examination, 873 cases were selected among the healthy children and adolescents aged 1-18 years who referred to the endocrinology clinic of Amirkola Children's Hospital for growth evaluation. After overnight fasting, early morning blood samples were obtained to measure the ALP level and other biochemical parameters using an automatic biochemical analyzer. Subjects were categorized by age, sex, and body mass index (BMI) values. The age groups were categorized as follows: 1-4 years, 5-8 years, 9-13 years, and 14-18 years.

**Results:**

There was a significant difference among the age and sex categories; on the contrary, there was no meaningful variation between the two groups categorized by BMI. The reference range for ALP was 474.14-517.71 U/L for children aged 1-4 years, 273.47-871.44 U/L for 5-8 years, 215.04-893.69 U/L for 9-13 years, and 228.9-739.22 U/L for 14-18 years. Also, significant positive correlation was found between ALP with length (P=0.000, r=0.134), weight (=0.04, r=0.073), phosphorus (P) (P=0.001, r=0.122), and alanine aminotransferase (SGPT) (P=0.000, r=0.142) respectively.

**Conclusion:**

This project's data established a reference interval for ALP in healthy children and adolescents, which will prepare a basis for diagnosis and monitoring liver- or bone-related disorders.

Alkaline phosphatase (ALP) activity presents in most body tissues, particularly in the small intestinal mucosa and the proximal convoluted tubule of the kidney, bone (osteoblasts), liver, and placenta, which is attached to membranes and cell surfaces. ALP converts phosphate esters in an alkaline environment to organic radicals and inorganic phosphates. Increased serum ALP activity originates from liver and bone; therefore, serum ALP measurement is essential in studying liver-biliary disorders and bone disease related to increased osteoblast activity (1, 2). The highest ALP activity has been found in the bone and ossifying cartilage of growing laboratory animals, indicating its role in precocious ossification (1, 3). In bone, ALP binds to type 1 collagen, preparing the skeletal matrix for the mineralization and hydrolysis of organic phosphates and transporting inorganic phosphates and calcium into the cell. It also inhibits pyrophosphate and other mineralization inhibitors by removing their phosphates (4-6). ALP is a marker for osteoblastic activity, and growing children have higher ALP levels (7-9). Even in some children, the ALP concentration may be 5 to 10 times than the adult reference interval.

The highest ALP levels are detected during the developmental phase of childhood and adolescence (3, 9). While the reference interval is entirely dependent on age and gender in childhood, most commercial assessment kits used in the clinical laboratory have only an adult reference range and no child-specific limits.Knowing the normal range of ALP in different genders and ages is very important in interpreting the various diseases that increase or decrease their level,such as in failure to thrive,liver diseases or bone disorders.On the other hand, ALP level can change in vitamin D insufficiency or deficiency. For example, Ahmed et al. established the reference intervals for ALP levels in Pakistani children aged 1 to 17 years. The population-specific reference intervals lead to an accurate understanding of the fluctuations in ALP activity with increase (10). Due to the fact that in the extensive studies have not been conducted to determine interval reference for children in Iran, we decided to determine the reference interval of ALP in healthy Iranian children aged 1-18 years.

## Methods

**Study population: **The total study population included 873 individuals aged 1 to 18 years. All healthy children aged 1-18 years who referred to the endocrinology clinic of Amirkola Children's Hospital were evaluated to monitor growth. Children were first asked a medical history, and then all were monitored for a full physical examination. Children with a history of known underlying diseases, including liver, kidney, heart, and bone diseases (such as rickets), were excluded from the study. Children who had abnormal physical examinations (including abnormal heart sound, enlarged liver or spleen, etc.) were excluded from the study for a complete examination. The children's height and weight were then measured and transferred to the related growth charts. Children whose height and weight fell below the 3% curve were also excluded from the study. The clinical charts for infants and older children (aged 2 to 20 years) are available in two sets. Set 1 has the outer limits of the curves at the 5th and 95th percentiles and set 2 has the outer limits of the curves at the 3rd and 97th percentiles. Pediatric endocrinologists use set 2 to assess the growth of children with special health care requirements. Infants (birth to 2 years) must be measured for length and the weight-for-age charts for infants must be plotted. At age 2 years and older, stature should be measured and the stature-for-age chart for children (2 to 20 years) must be plotted. Children's weight was measured with a digital Balas scale made in Iran with an error of 0.2 grams. Children's height was estimated with a stadiometer. Then, body mass index (BMI) was calculated based on the formula of weight (in kilograms) divided by height (in meters) in the power of two and then transferred to the BMI chart. Body weight (kg) was measured on a Seca balance scale (to the nearest 0.1 kg). To measure the height, a Harpenden fixed stadiometer was used (to the nearest 0.1 cm). 

To examine the relationship between BMI and ALP, normal BMI was considered (a normal BMI or 5-85% of BMI curve means that 80 percent of the 2 to 20 years population are in this part of the BMI curve). To monitor growth, all children studied were examined in a reference laboratory. These tests included serum levels of ALP, calcium(Ca), phosphorus (P), 25-OH Vit D (VD), urea, creatinine (Cr), aspartate aminotransferase (SGOT), alanine aminotransferase (SGPT), T4, and TSH. These tests are needed to monitor children's growth, and no extra blood samples were taken from children. Children with abnormal laboratory results such as decreased calcium (≤ 8.5 mg/dl), decreased phosphorus (< 4 mg/dl) (3). increased urea (> 45 mg/dl) (3, 11) and creatinine (adjusted for age), and elevated SGPT (> 40 U/l) and SGOT (> 37 U/l)(12) were excluded from the study. Demographic information, height, weight, and test results for each child were entered and documented in a research form (checklist) to collect and analyze the data.

**Sampling and biochemical analytes measurement: **After overnight fasting, venous blood samples were collected in early morning (10 ml) from the children. After clot formation, the serum was separated by centrifugation at 3000 rpm for 15 minutes at room temperature. Serum was transferred to a microtube and stored at 70 °C to measure biochemical factors. Serum SGPT, SGOT, ALP, urea and Cr were measured by a Japanese Hitachi 917 biochemical auto analyzer using an AUDIT kit. T4 and TSH were assessed using the electrochemiluminescence technique with COBAS e400 (Elecsis kit , Roche company) , and calcium and phosphorus were measured using the AUDIT kit's photometric method. The level of VD in the samples was measured using the chemiluminescence technique with Liaison analyser ( Diasorin Liaison Immunodiagnostic C.L.I.A). VD≥20ng/ml was considered sufficient, and less than that was considered low level of VD (13). 

ALP level was measured using statistical methods, the lower and upper limits of ALP were determined. The obtained data were analyzed using SPSS software Version 18. The ALP kit limit ranged from 5 U/L to the linear limit of 1000 U/L. No difference was observed between the results obtained from the bionic diagnostic kit and other commercial kits, comparing the kits' accuracy (correlation coefficient (r)=0.996). The mean±sd of intraassay for ALP was 449.35±5.01 (% CV=1.11). The mean±sd of interassay for ALP was 449.62±5.45 (% CV=1.21).

**Statistical analysis: **All variables were analyzed by IBM SPSS Statistics software, Version 18·0, and reported as mean ± standard deviation (SD). For evaluating the normality of data distribution, Kolmogorov–Smirnov test was used. Student's t-test and one-way analysis of variance (ANOVA) were employed for the comparison of values. Pearson's test measured the correlations between ALP and other parameters. A p-value less than 0·05 was considered as a significant variation. This study was approved by the Ethics Research Committee of Babol University of Medical Sciences (code: IR.MUBABOL.HRI.REC.1400.092). 

## Results

In this study, the records of 873 children and adolescents aged 1 to 18 years were examined. The anthropometric and metabolic characteristics of the study population are given in table 1. The results were expressed as mean ± standard deviation in four age groups. According to the table 1, there was a significant difference in levels of Cr (P=0.00), P (P=0.00), ALT (P=0.01), AST (P=0.00), BMI (P=0.00), and ALP (P=0.00) among the four age groups. However, Vit D, T4, TSH, Ca, and urea levels did not significantly differ between the four groups (p>0.05). Reference values for measured ALP in the current study are summarized in table 2. 

**Table 1:** Laboratory characteristics in study groups, separated by age

	**GroupI** **(1-4years)** **(n=140)**	**GroupII** **(5-8years)** **(n=289)**	**GroupIII** **(9-13years) (n=308)**	**GroupIV** **(14-18years)** **(n=136)**	**P-value**
	**Mean±SD**	**Mean±SD**	**Mean±SD**	**Mean±SD**	
Urea (mg/dL)	24.66±7.92	23.83±7.12	23.41±7.01	25.44±8.01	0.21
Cr (mg/dL)	0.49±0.08	0.57±0.08	0.65±0.09	0.75±0.18	0.000
Ca (mg/dL)	9.87±7.71	9.83±0.37	9.78±0.36	9.85±0.35	0.145
P (mg/dL)	5.32±0.52	5.11±0.56	4.93±0.71	4.33±0.81	0.000
Vitamin D (ng/mL)	21.29±11.41	20.07±12.60	19.64±12.39	23.07±12.66	0.31
ALT (U/L)	16.01±5.36	17.82±8.05	18.04±8.25	19.94±7.77	0.017
AST (U/L)	29.23±6.23	26.19±6.01	24.11±5.69	24.02±6.55	0.00
T4 (μg/dL)	9.56±5.62	8.97±1.61	8.79±5.58	7.80±0.97	0.14
TSH (mIU/L)	2.53±1.37	2.7±1.31	2.6±1.19	2.23±0.98	0.17
ALP (U/L)	495.93±130.36	558.00±144.07	567.94±170.53	380.47±173.86	0.000
BMI (kg/m^2^)	16.35±9.16	17.58±8.88	19.80±5.35	24.58±5.03	0.000

All findings are given as mean ±sd. * P-value notes to the comparison of each parameter between age groups. SD= standard deviation; BMI= Body mass index; T4= Thyroxine; TSH= Thyroid stimulating hormone; Ca= Calcium; P= phosphorus; Cr= creatinine; ALP=Alkaline phosphatase; ALT= Alanine aminotransferase; AST= Aspartate aminotransferase.

As shown in table 2, the mean±sd of ALP of the 1-4 years was 495.93±130.36 U/L with a reference interval of 474.14-517.71 U/L. The 5-8 years' ALP value was 558.00±144.07 U/L (mean±sd), and the obtained reference range was 273.47-871.44 U/L. The 9-13 years ALP level reference ranges were 215.04-893.69 U/L, with the mean±sd of 567.94±170.53 U/L. For 14-18years, the mean±sd and the obtained reference ranges were 380.47±173.86 U/L and 228.9-739.22 U/L. Respectively, with the reference interval of <18 children: 180-1200u/l, men: 80-306u/l, and women: 64-306. Figure 2 shows serum ALP levels by age and gender. According to table 3, out of 873 recorded children, 557 (63%) children were in the BMI normal range (between 5th to 85th percentile). Of these, 281 (50.4%) were girls, and 276 (49.6%) were boys. As shown in table 4, the normal changes of BMI value are shown as mean±SD in the four age groups. The mean of normal BMI was 15.4351±0.99 in the age group of 1-4 years, 15.3410±1.17 in the age group of 5-8 years, 17.0993±2.01 in the age group of 13-19 years, and 20.2795±2.4 in the age group of 14-18 years. Table 5 shows the correlation between ALP with children's age and normal BMI. According to table 5, there was a significant correlation between the age of children with normal BMI and BMI values (5th to 8th percentile) (p<0.001). However, no significant correlation was observed between children's age with normal BMI and ALP levels (p>0.01). Also, no significant correlation was observed between BMI values in children with normal BMI and ALP levels (p>0.01).

**Table 2:** Reference intervals for serum ALP levels in healthy children aged 1-18 years

**ALP** **(years)**	**N**	**F/M**	**Mean±SD**	**Reference interval**		**95% Confidence Interval for Mean**		**Minimum**	**Maximum**
				**Lower Bound**	**Upper Bound**	**Lower Bound**	**Upper Bound**		
1-4	140	65/75	495.93±130.36	252.96	770.25	474.1444	517.7128	179.00	926.00
5-8	289	119/170	558.00±144.07	273.47	871.44	541.3222	574.6847	149.00	996.00
9-13	308	146/162	567.94±170.53	215.04	893.69	548.8247	587.0649	129.00	1200.00
14-18	136	88/48	380.47±173.86	228.9	739.22	319.8065	441.1347	153.00	792.00

All results are noted as mean ±sd. SD= standard deviation; ALP=Alkaline phosphatase; F= Female; M= Male

**Table 3:** Analysis of children 1 to 18 years in normal BMI values *(****between the 5th percentile and 85th percentile****)*

Valid	Frequency	Percentage	Valid Percentage	Cumulative Percentage
**Female**	281	50.4	50.4	50.4
**Male**	276	49.6	49.6	100.0
**Total**	557	100.0	100.0	

All findings are given as percentages and frequencies.

**Table 4:** Changes of BMI (5th to 85th percentile) in four age groups

Age (year)	N	Mean ± SD	95% Confidence Interval for Mean		Minimum	Maximum
			Lower Bound	Upper Bound		
**1-4**	103	15.4351±0.99	15.2400	15.6302	13.63	17.85
**5-8**	160	15.3410±1.17	15.1570	15.5250	13.61	18.63
**9-13**	177	17.0993±2.01	16.8058	17.4035	13.99	22.52
**14-18**	117	20.2795±2.4	18.9908	21.5681	14.88	24.39
**Total**	557	16.2141±1.93	16.0355	16.3928	13.61	24.39

All results are presented as mean ±sd.

**Table 5:** Correlation of serum alkaline phosphatase levels with age and BMI

		age	BMI	ALP
**Age**	Pearson Correlation	1	0.525^**^	0.084
	Sig. (2-tailed)		0.000	0.072
	N	557	557	557
**BMI**	Pearson Correlation	0.525^**^	1	0.036
	Sig. (2-tailed)	0.000		0.443
	N	557	557	557
**ALP**	Pearson Correlation	0.084	0.036	1
	Sig. (2-tailed)	0.072	0.443	
	N	557	557	557

**. Correlation is significant at the 0.01 level (2-tailed)

BMI= Body mass index; ALT= Alanine aminotransferase.

**Figure 1:**
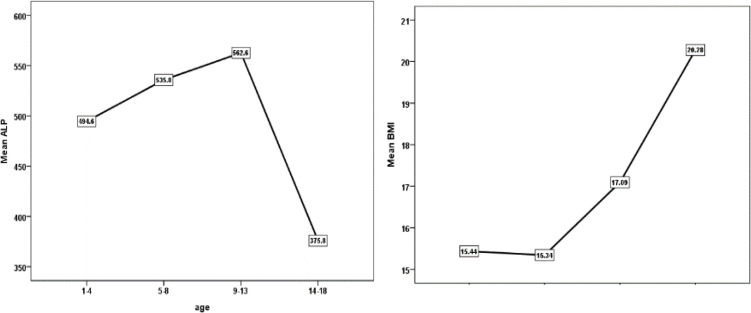
The change of ALP of the children with normal BMI in 4 age groups

**Figure 2:**
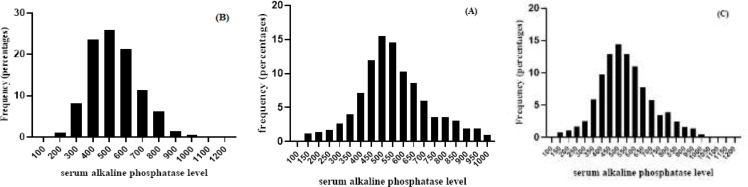
Histogram of the serum ALP values for all reference individuals. A) healthy boys (skewness:0.62; kurtosis:0.78, mean±SD: 555.23±166.651 ); B) healthy girls (skewness:0.3 ; kurtosis:0.18, mean±SD: 528.15±150.53 ); C) total of 873 healthy children (skewness:0.44; kurtosis:0.38, mean±SD:542.88±159.97). ALP=Alkaline phosphatase

Figure 1 shows the change of ALP of the children with normal BMI in 4 age groups. According to figure 1, mean levels of ALP in the children with BMI normal value increased in 4 age groups with increasing age and reached its maximum activity in 9 to 13 years (average 562.6 U/L) and significantly decreased in 14 to 18 years old. (average 375.8 U/L). Also, the average BMI changes in four age groups of children with normal BMI gradually increased with age and reached its maximum in puberty and adolescence (average 20.28 in the age group of 14 to 18 years). Figure 2 (A-C) showed how serum alkaline phosphatase levels were distributed in girls, boys,and the general population. According to figure 2, the results showed that ALP levels were significantly different between the male and female groups (P=0.01).

## Discussion

According to the Clinical and Laboratory Standards Institute (CLSI) guideline, 873 children and teenagers aged 1 to 18 years in the desired clinic were selected in this study. Their serum ALP levels were assessed to establish the biological reference interval. Following the children's growth characteristics during infancy, childhood, and adolescent age, all subjects were distributed into the following age groups: 1-4, 5-8, 9-13, and 14-18 years Using statistical analysis, we found a significant statistical difference between the above age groups. As a result, a distinct biological reference value was specified for these groups. From these age groups, we determined the biological reference values of 474.14-517.71 U/L for children aged 1-4 years, 273.47-871.44 U/L for 5-8 years, 215.04-893.69 U/L for 9-13 years, and 228.9-739.22 U/L for 14-18 years. The clinical laboratory participates in providing information for the clinical diagnosing and monitoring of various disorders. Trusty reference intervals are essential for minimizing incorrect diagnoses for several diseases; therefore, the clinical laboratory requires preparing suitable reference intervals, as an evaluation scale, to diagnose disease (14-17). ALP is one of the routine tests in the medical diagnostic laboratory, which the physicians mainly request to check for liver and bone disorders (18). ALP isoenzymes present in various body tissues and catalyze the same reaction, including the hydrolysis of organic phosphate esters (19). These isoenzymes are present in small amounts in the intestine, placenta, bone, kidney, and liver organs (20-22). Serum ALP is routinely inquired about a part of the liver function tests (LFTs) panel and bone disorder analyses. More than 80% of serum ALP concentration is due to the enzyme's release from the liver and bone (20, 23, 24). Thus, the majority of ALP concentrations are commonly derived from obstructed liver or growing bone. Clinical investigations with the evaluation of bone-specific ALP levels showed that 77-89% of the total serum ALP concentration in children are of bone origin. Children experience bone growth, and therefore the amount of bone ALP in their serum increases (25). Low serum ALP concentrations are less common and are mostly linked to a nutritional deficiency or genetic state, and normal values of ALP can be influenced by age, sex, growth, or hormonal status (26, 27). 

Previous studies indicated that metabolic syndrome is an important cause of elevated ALT levels in adolescents. Oliveira et al. exhibited that the increase in waist circumference and BMI are associated with higher ALT levels (28). In the current study, we found that the average BMI change gradually increases with age in the four age groups of children with normal BMI and reaches its maximum in puberty and adolescence compared to childhood. Furthermore, the average ALP level in children with BMI normal values increased with increasing age in four age groups and reached its maximum activity in 9 to 13 years. However, in 14 to 18 years, the mean ALP level significantly decreased. This could be due to possible hormonal changes during puberty, decreased metabolism, and the gradual epiphyseal plate closure (29). Except for age-stratified intervals, ALP also required gender-separated reference intervals. So, we subsequently divided children into two distinct groups of boys and girls. ALP showed a distinct gender dependency during childhood. ALP has also been recognized to reflect the height growth rate. A marked reduction of ALP, especially in females aged 14 to 18 years, might reflect a decreased height growth. These results were higher than Li et al.’s study. They determined age-and gender-specific reference intervals for ALP and other liver biochemical parameters in 1394 healthy Han children aged 2 to 14 years. The reference interval for children aged 2-3 years was 160-376 U/L, for children aged 4-9 years was 143-362 U/L, for children aged 10-12 years was 116-426 U/L, and for children aged 13-14 years was 48-232 U/L (30). In another study, Turan et al. evaluated serum ALP levels in 1741 healthy children. There was no difference in serum ALP concentration between girls and boys up to puberty period. Higher serum levels of ALP were observed at 10 to 11 years in girls and 12 to 17 years in boys. On the contrary, these levels began to decrease after 12 and 16 years in girls and boys, respectively (25). 

Marwaha et al. described the reference interval of some biochemical parameters including ALP in a total 3700 healthy Asian-Indian children aged 6–17 years. According to their findings, ALP was notably higher in boys than girls with highest levels at the age of 8 and showed an inverse association with age(31) . Nevertheless, the reports related to ALP reference intervals for healthy children in the United States, China, South Korea, Italy, and Brazil have been published, and our findings are from the northern region in Iran. According to these findings, the data's differences may be described by ethnicity, eating habits, and geographical region. Moreover, the differences in measurement methods, instruments, reagents, or the number of desired groups may change the outcome (30, 32-36). As a result, it is essential to establish a reference interval for the local population's medical laboratory. However, one of the limitations of this study is that the children's pubertal status did not check. The increase in ALP during pubertal ages reflects the rapid growth in puberty. There were differences between boys and girls during this period. 

Our study is the first establishing reference intervals for ALP parameter in 873 Iranian children and teenagers (aged 1 to 18 years) following the children's growth characteristics during infancy, childhood, and adolescent age. Based on this study's results, we hope that this reference interval for serum ALP levels will be beneficial in diagnosing and monitoring patients with abnormal ALP concentration. Determination of reliable reference intervals for ALP will permit a more accurate evaluation of changes in serum levels of ALP in rickets or other bone disorders. It is recommended that future studies with higher sample sizes be performed to determine the reference intervals of other biochemical parameters.
